# Building a Global Research Network for Fair, Accountable, Interpretable, and Responsible AI in Emergency Care: Protocol for a FAIR-EC Study

**DOI:** 10.2196/74202

**Published:** 2026-08-04

**Authors:** Chuan Hong, Jonathan Chong Kai Liew, Jaeyong Yu, Tomás Barry, Audrey L Blewer, Daniel M Buckland, Tianrun Cai, Won Chul Cha, Bibhas Chakraborty, Wei Chen, Jun Cheng, Shu-Ling Chong, Therese Djärv, Arul Earnest, Matthew Engelhard, Xiuyi Fan, Mengling Feng, Jean Feng, Huazhu Fu, Wilson Wen Bin Goh, Benjamin A Goldstein, Jessica Gronsbell, Andrew Fu Wah Ho, Kendall Ho, Taku Iwami, Anjni Joiner, Aaron Kornblith, Siqi Li, Shir Lynn Lim, Molei Liu, Zhenghong Liu, Lei Lu, Yuan Luo, Yih Yng Ng, Yilin Ning, Yohei Okada, Ju Ok Park, Yu Rang Park, Junaid Razzak, Yuzeng Shen, Fahad Javaid Siddiqui, Peter A D Steel, Kenneth Boon Kiat Tan, Salinelat Teixayavong, Bella Vakulenko-Lagun, Joao Ricardo Nickenig Vissoci, Grzegorz Waligora, Fei Wang, Haibo Wang, Haoyuan Wang, An-Kwok Ian Wong, Feng Xie, Jie Yang, Yiye Zhang, Doudou Zhou, Li Zhou, Tingting Zhu, Robert Neumar, David Page, Roger Vaughan, Marcus Eng Hock Ong, Nan Liu

**Affiliations:** 1 Department of Biostatistics and Bioinformatics, School of Medicine, Duke University, Durham, NC, United States; 2 Perelman School of Medicine, University of Pennsylvania, Philadelphia, PA, United States; 3 Hallym University, Chuncheon, Republic of Korea; 4 School of Medicine, University College Dublin, Dublin, Ireland; 5 Department of Family Medicine and Community Health, School of Medicine, Duke University, Durham, NC, United States; 6 Department of Population Health Sciences, School of Medicine, Duke University, Durham, NC, United States; 7 Health Services Research and Population Health, Duke–National University of Singapore (NUS) Medical School, Singapore, Singapore; 8 Department of Emergency Medicine, University of Wisconsin - Madison, Madison, Wisconsin, United States; 9 VA Boston Healthcare System, , Boston, MA, United States; 10 Brigham and Women’s Hospital, Boston, MA, United States; 11 Samsung Medical Center, Seoul, Republic of Korea; 12 School of Biomedical Engineering, The University of Sydney, Sydney, Australia; 13 Institute for Infocomm Research (I2R), Agency for Science, Technology and Research, Singapore, Singapore; 14 KK (Kandang Kerbau) Women's and Children's Hospital, Singapore, Singapore; 15 Karolinska University Hospital, Stockholm, Sweden; 16 Karolinska Institutet, Stockholm, Sweden; 17 School of Public Health and Preventive Medicine, Monash University, Melbourne, Australia; 18 Lee Kong Chian School of Medicine, Nanyang Technological University, Singapore, Singapore; 19 College of Computing and Data Science, Nanyang Technological University, Singapore, Singapore; 20 Saw Swee Hock School of Public Health, National University of Singapore, Singapore, Singapore; 21 Institute of Data Science, National University of Singapore, , Singapore, Singapore; 22 Department of Epidemiology and Biostatistics, University of California, San Francisco, San Francisco, CA, United States; 23 Institute of High Performance Computing, Agency for Science, Technology and Research, Singapore, Singapore; 24 Nanyang Technological University, Singapore, Singapore; 25 Imperial College London, London, United Kingdom; 26 Department of Statistical Sciences, University of Toronto, Toronto, ON, Canada; 27 Duke–National University of Singapore (NUS) Medical School, Singapore, Singapore; 28 Singapore General Hospital, Singapore, Singapore; 29 Centre for Population Health Research and Implementation, SingHealth, Singapore, Singapore; 30 Department of Emergency Medicine, University of British Columbia, Vancouver, British Columbia, Canada; 31 Department of Preventive Services, School of Public Health, Kyoto University, Kyoto, Japan; 32 Department of Emergency Medicine, Duke University, Durham, NC, United States; 33 Department of Emergency Medicine, University of California, San Francisco, San Francisco, CA, United States; 34 Centre for Biomedical Data Science, Duke-NUS Medical School, 8 College RoadSingapore, S169857, Singapore, 65 66016503; 35 National University Heart Centre Singapore, Singapore, Singapore; 36 Department of Biostatistic, Columbia University’s Mailman School of Public Health, New York, NY, United States; 37 School of Life Course & Population Sciences, King's College London, London, United Kingdom; 38 Department of Preventive Medicine, Northwestern University Feinberg School of Medicine, Chicago, IL, United States; 39 College of Medicine, Hallym University, Chuncheon, Republic of Korea; 40 Hallym University Dongtan Sacred Hospital, Hwaseong-si, Republic of Korea; 41 Department of Biomedical Systems Informatics, Yonsei University College of Medicine, Seoul, South Korea; 42 New York Presbyterian-Weill Cornell Medicine, New York, NY, United States; 43 Center of Excellence for Trauma and Emergencies, Aga Khan University, Karachi, Pakistan; 44 University of Haifa, Haifa, Israel; 45 Gdańsk Medical University, Gdansk, Poland; 46 Wroclaw Medical University, Wroclaw, Poland; 47 Weill Cornell Medicine, New York, NY, United States; 48 Research Centre of Big Data and Artificial Intelligence for Medicine, The First Affiliated Hospital, Sun Yat-sen University, Guangzhou, China; 49 Center for Health AI and Synthesis of Evidence (CHASE), Department of Biostatistics, Epidemiology and Informatics, Perelman School of Medicine, University of Pennsylvania, Philadelphia, PA, United States; 50 The Graduate Group in Applied Mathematics and Computational Science, School of Arts and Sciences, University of Pennsylvania, Philadelphia, PA, United States; 51 Section of Pulmonary and Critical Care Medicine, Department of Medicine, Durham VA Medical Center, Durham, NC, United States; 52 Department of Surgery, Division of Computational Health Sciences, University of Minnesota, Minneapolis, MN, United States; 53 Division of Pharmacoepidemiology and Pharmacoeconomics, Department of Medicine, Brigham and Women’s Hospital, Harvard Medical School, Harvard University, Boston, MA, United States; 54 Broad Institute of MIT and Harvard, , Cambridge, MA, United States; 55 Harvard Data Science Initiative, Harvard University, Cambridge, MA, United States; 56 Department of Statistics and Data Science, National University of Singapore, Singapore, Singapore; 57 Harvard Medical School, Boston, MA, United States; 58 University of Oxford, Oxford, United Kingdom; 59 Department of Emergency Medicine, Michigan Medicine, University of Michigan-Ann Arbor, Ann Arbor, MI, United States; 60 University of Pennsylvania, Philadelphia, PA, United States; 61 NUS Artificial Intelligence Institute, National University of Singapore, Singapore, Singapore

**Keywords:** emergency care, emergency medicine, pre-hospital Intensive Care Unit (ICU), federated learning, research network

## Abstract

**Background:**

The current landscape of emergency care (EC) is marked by high demand, leading to issues such as emergency department boarding, overcrowding, and subsequent delays that impact the quality and safety of patient care. Integrating data science into EC can enhance decision-making with predictive, preventative, personalized, and participatory approaches. However, gaps in adherence to fairness, accountability, interpretability, and responsibility are evident, particularly due to barriers to data-sharing, which often result in a lack of transparency and robust oversight in these applications.

**Objective:**

The FAIR-EC (Fair, Accountable, Interpretable, and Responsible-Emergency Care) collaboration adapts the existing Fair, Accountable, Interpretable, and Responsible principles to address emerging challenges as data science integrates with EC. This initiative aims to transform EC by establishing ethical artificial intelligence standards specifically tailored for this integration. By bridging the gap between EC professionals, data scientists, and other stakeholders, the collaboration promotes international cooperation that leverages advanced data science techniques to enhance EC outcomes across different care settings.

**Methods:**

We propose a federated research design to analyze extensive datasets from various global institutions without compromising patient privacy. This approach transforms epidemiological research with advanced data science techniques, emphasizing the harmonization of data for comprehensive analyses across different health care systems.

**Results:**

The FAIR-EC initiative has facilitated the identification and harmonization of datasets from diverse geographical regions, enabling the examination of regional variations in EC practices. As of paper submission, participating sites have identified retrospective EC datasets totaling >2 million records (eg, Duke Health >400,000 and Singapore General Hospital >1.7 million records). Initial projects have demonstrated feasibility and operational readiness, including implementation of federated workflows and ongoing development of a federated scoring system, cross-site evaluation, and adaptation of association studies and predictive models across various regions. Cross-site harmonization and pilot analyses are underway (with local ethics approvals in progress), and first multisite results are expected to be submitted in mid-late 2026, with additional project-level publications anticipated in 2027. These efforts highlight the feasibility of leveraging advanced data science techniques to address the complexities of EC while preserving patient privacy without centralizing individual-level data. This project was funded from September 1, 2022, to August 31, 2023.

**Conclusions:**

FAIR-EC integrates data science ethically and effectively into EC, addressing challenges such as fragmented data, real-time handoffs, and public health crises. Its federated design harmonizes diverse data streams while preserving privacy, and its emphasis on ethical artificial intelligence aligns with the dynamic nature of EC. Despite challenges in data variability and system complexity, FAIR-EC establishes a strong foundation for innovation in global EC.

## Introduction

### Current Status and Demands of Emergency Care

Emergency care (EC) involves the rapid diagnosis, evaluation, and treatment of patients with acute illness or injury and decompensated chronic illness, spanning prehospital care (eg, ambulances), emergency departments (EDs), intensive care units, and beyond (eg, operating rooms and procedural units). Its critical role is to ensure timely diagnosis and interventions that reduce morbidity and mortality across medical, surgical, and psychiatric emergencies [1].

Global demand for EC is surging, straining health care systems with ED overcrowding, delayed admissions, and prolonged wait times [2]. These challenges stem from factors such as aging populations, longer lifespans, and rising care expectations, creating a mismatch between demand and system capacity [3,4]. In the United States, ED overcrowding is primarily driven by inpatient boarding, where admitted patients remain in the ED due to inadequate inpatient capacity, rather than a significant increase in ED visits or hospitalizations over the past decade.

To address these issues, innovative solutions are needed to improve efficiency and decision-making across the entire EC continuum, from initial health system contact to postacute recovery. This includes optimizing prehospital and ED management, expediting inpatient admissions, and integrating data across hospital wards, skilled nursing facilities, rehabilitation centers, and outpatient follow-up clinics. Leveraging electronic health records (EHRs) and complementary data sources (such as secure messaging, wearable devices, and home monitoring) systemically enables better coordinated, comprehensive, and longitudinal patient care.

### Integrating Data Science and Artificial Intelligence in EC

Clinical decision-making in EC has traditionally relied on physicians’ experience, supplemented by heuristic decision-support tools. However, in high-pressure environments such as overcrowded EDs, this approach can be inconsistent due to variations in adherence to evidence-based frameworks. Artificial intelligence (AI)–based clinical support algorithms offer a solution by synthesizing vast amounts of data, reducing cognitive biases, and ensuring more standardized, data-driven decisions. The widespread adoption of EHRs in EC presents opportunities to enhance decision-making through AI-driven analysis [5]. By leveraging structured data and real-time monitoring, AI models can predict conditions such as sepsis before clinical symptoms appear, optimizing workflows and improving patient outcomes [6] though their real-world implementation remains limited [7-9].

### Gaps and Opportunities

Current EC methodologies lack full integration of ethical AI principles, leading to oversimplified decision-making that fails to address inherent biases, disproportionately affecting marginalized populations [10]. Additionally, disparities in access to AI tools hinder low-resource EDs from benefiting from association and predictive analytics, and real-time decision support, widening global health inequities [11]. Transparency and interpretability remain major challenges, limiting clinician trust and adoption [10,12,13]. Insufficient accountability and regulatory oversight further risk the ethical and responsible use of AI in EC [14]. Ethical concerns, such as patient autonomy and privacy, also require more rigorous safeguards [15]. International, interdisciplinary collaboration presents a critical opportunity to bridge these gaps [16,17]. By integrating expertise from data scientists, EC professionals, ethicists, and patient advocates, one can develop scalable, transparent, and interpretable AI models to ensure equitable care and set new global standards for EC.

### Ethical AI Standards in EC

Ethical AI integration in EC faces significant technical and systemic barriers, including data accessibility [18], integration challenges, privacy concerns, incomplete data, and real-time processing constraints [15,19,20]. To address these challenges, AI implementation must adhere to the core principles of the Fair, Accountable, Interpretable, and Responsible framework (Multimedia Appendix 1 [21-30]). “Fairness” ensures equitable outcomes, preventing algorithmic bias and promoting equal care across diverse patient populations [23]. Methods such as data rebalancing [31], threshold recalibration [32], and performance monitoring [33] can help mitigate biases. “Accountability” requires that AI developers, clinicians, and regulators ensure justifiable algorithmic decisions and establish clear protocols for addressing errors or biases, fostering transparency and trust [34-37]. “Interpretability” is crucial for clinical adoption, allowing health care providers to understand AI-driven recommendations and ensuring that AI supports rather than dictates clinical care [12,24,25]. “Responsibility” entails the ethical use of AI, ensuring patient safety, privacy, and dignity while enhancing decision-making [26,38-41].

### FAIR-EC Network

The FAIR-EC (Fair, Accountable, Interpretable and Responsible-Emergency Care) network is built on the principle that international collaboration is key to developing ethical, scalable data science techniques in EC. Given the variability in patient demographics, health care infrastructure, and resources across regions, evaluating data-driven solutions in diverse settings—including low-resource environments—is essential. The Pan-Asian Resuscitation Outcomes Study [42] underscores disparities in EC data availability, highlighting the need for adaptable techniques. By uniting emergency professionals and data scientists, FAIR-EC ensures solutions are both theoretically sound and effective in real-world applications.

### Master Data Representation

A key aspect of this framework is the master data representation, which facilitates interoperability across diverse EC datasets. This is achieved through the adoption of standardized data models, including the Patient-Centered Outcomes Research Network common data model, Observational Medical Outcomes Partnership, and Fast Healthcare Interoperability Resources standards, which provide structured, interoperable representations of patient records, diagnoses, interventions, and outcomes. Additionally, ontology mapping and terminology harmonization using the Unified Medical Language System and SNOMED CT (Systematized Nomenclature of Medicine – Clinical Terms) ensure that medical concepts are consistently interpreted across institutions.

To further unify data across institutions, FAIR-EC leverages natural language processing [43] and large language models (LLMs) [44] for cross-institutional data harmonization. BERT (Bidirectional Encoder Representations From Transformers)-based models are used for entity recognition and concept extraction from clinical notes, while graph convolutional networks (GCN) are used for knowledge graph-based patient stratification. Additionally, LLM-enhanced data augmentation improves data imputation strategies and supports missingness-aware predictions. FedIMPUTE, a federated-transfer imputation method, is specifically designed to address missing data across institutions, enhancing model robustness and consistency [45].

### Federated Learning and Transfer Learning for Model Development

Federated learning [46] enables decentralized model training without sharing raw patient data, preserving privacy while allowing for cross-institutional collaboration. Key implementations include FedScore, a federated scoring system for association and predictive modeling, and FedFML, a federated meta-learning approach that tailors models to individual institutions while leveraging global insights.

The Score for Emergency Admission Prediction project evaluates the generalizability of Singapore’s inpatient admission prediction models across international sites, ensuring their applicability beyond their original development context. Meanwhile, TRACER (Dynamic Transfer Learning) is designed to continuously adapt models as new data becomes available, ensuring they remain relevant and effective in evolving clinical environments.

## Methods

### Study Design and Setting

FAIR-EC is based on a federated research design, a strategic approach that enables the collaborative analysis of data from diverse institutions without necessitating the sharing of individual patient-level data, while applying transfer learning techniques [33,47-49]. While this avoids the centralization of data across different countries or regions, it is important to note that within each participating site, researchers still collect and store private and confidential data on individuals. This approach ensures the utmost respect for security while leveraging the collective strength of data to uncover insights that can significantly improve EC practices. Additionally, the federated learning framework adopted by the FAIR-EC study does not introduce new security risks, aligning with current best practices in AI to safeguard data integrity and confidentiality. Our federated learning framework is communication-efficient and easy to implement [50,51]. Therefore, even in low-resource settings, they can still implement federated learning algorithms and participate in this collaboration without requiring advanced hardware.

The collaboration will be conducted across various participating institutions worldwide, showcasing a commitment to international collaboration. By adopting this federated framework, FAIR-EC aims to foster a comprehensive understanding of EC dynamics on a global scale, facilitating the development of scalable, equitable, and effective data science solutions within the realm of EC.

### Core Study Definition and Scope

FAIR-EC functions both as a collaborative research network and a unified study protocol. At its core, this study aims to evaluate the feasibility, validity, and fairness of federated, interpretable machine learning models for key EC tasks such as admission prediction, triage, and discharge planning across diverse international sites. The primary outcomes include predictive performance (eg, area under the receiver operating characteristic curve [AUROC] and calibration), equity across subgroups using fairness metrics such as the group-conditional concordance index, and technical feasibility as measured by successful implementation of federated workflows, participation rates, and data completeness. Secondary outcomes focus on operational utility, user trust, and robustness under data shift or incompleteness. Specific analytic modules and modeling frameworks that address these outcomes are introduced in later sections of the protocol. In contrast, spin-off or locally initiated projects that focus on narrower clinical questions or extended data modalities will not be considered part of the core study, although they may build on FAIR-EC’s infrastructure and adhere to its principles.

### Participating Sites and Study Cohorts

FAIR-EC will use datasets originating from a broad geographical spectrum, including regions and countries from Asia, Australasia, North America, Europe, and the Middle East. These datasets are critical to understanding the global landscape of EC, providing insights into regional practices, outcomes, and challenges. Within each participating site, we target a wide-ranging population of patients receiving EC, with inclusion criteria designed to capture a broad spectrum of emergency presentations. Exclusion criteria will be carefully defined to ensure clarity and relevance of the data, focusing on removing cases that do not meet the EC criteria or lack sufficient data for meaningful analysis. Data collection will be conducted in a site-specific manner, tailored to meet the unique needs and capabilities of each participating location to ensure the accuracy and applicability of the data obtained.

Given the broad scope of FAIR-EC, specific inclusion and exclusion criteria, variable sets, and analytic strategies vary across individual projects and participating sites. Each project within the FAIR-EC network is required to define its analytic cohort, outcomes, modeling strategy, and validation metrics in detail through locally approved institutional review board (IRB) protocols. While we do not prescribe a single patient-level definition or analytic pipeline across all studies, we ensure consistency through harmonization guidance, cross-site code templates, and shared evaluation principles. As an illustrative example, one active project evaluates hospital admission prediction across three Duke hospitals using 2019 ED visits. The cohort includes adult ED encounters with available demographics, vital signs, and comorbidity data (eg, pulse, blood pressure, oxygen saturation, acuity level, and *ICD* [*International Statistical Classification of Diseases*]-based comorbidity scores). The primary outcome is inpatient admission, and the project compares federated imputation approaches against local and pooled baselines, evaluating mean squared error and AUROC. Similar project-specific protocols are under development at other institutions and will follow comparable standards for cohort definition, model comparison, and validation. Details for each will be introduced in subsequent sections or appendices.

### Interdisciplinary Collaboration Mechanisms

**Figure 1.**
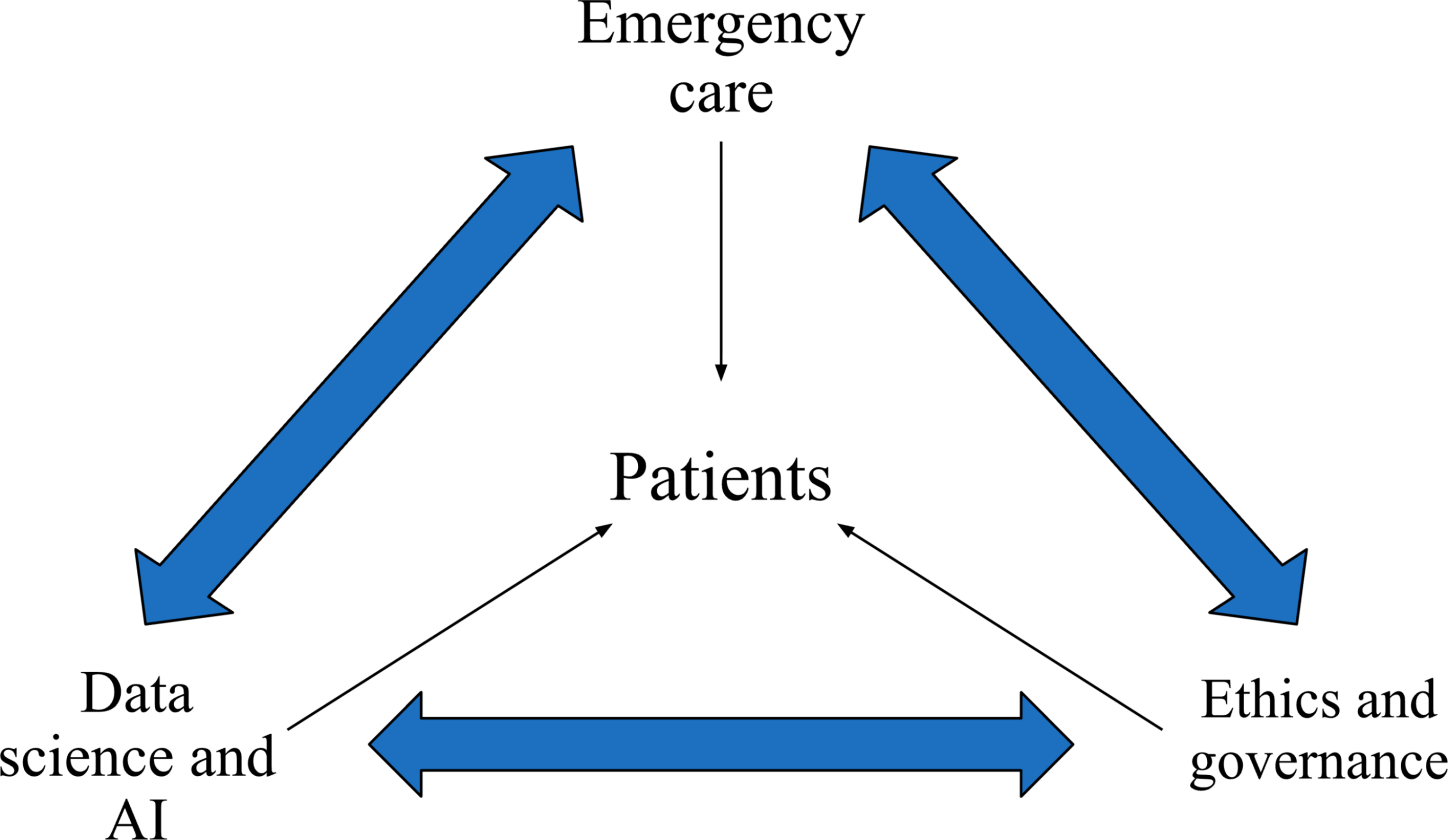
Interdisciplinary interaction mechanisms centered on the patient for collaboration in FAIR-EC, leveraging data across the entire episode of care—from initial health system contact to return to baseline or a new steady state. AI: artificial intelligence; FAIR-EC: Fair, Accountable, Interpretable, and Responsible-Emergency Care.

To facilitate interdisciplinary collaboration, a multifaceted infrastructure (Figure 1) will be established, encompassing emergency physicians and other EC providers, data scientists, social scientists, ethicists, patients, community supporters, and other relevant stakeholders. By establishing the mechanisms for interdisciplinary collaboration, communication, and iterative feedback, FAIR-EC aims to create a dynamic environment where innovative solutions are developed through the synergy of diverse expertise.

### Collaborative Platforms

**Figure 2.**
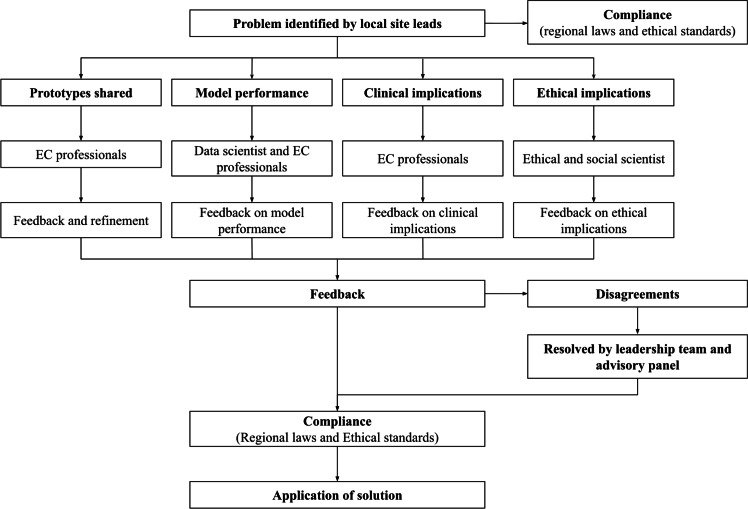
Iterative feedback mechanisms and stakeholder engagement. EC: emergency care.

A digital collaboration platform (Figure 2) will be used to foster communication and project management among the interdisciplinary team members. This platform will support document sharing, project tracking, and real-time discussions, ensuring that all participants, regardless of their geographic location, can contribute effectively to this project.

### Cross-Disciplinary Teams

This study will organize participants into cross-disciplinary teams, each tasked with addressing specific aspects of this project. These teams will blend the expertise of EC professionals, data scientists, epidemiologists, biostatisticians, and ethicists to ensure that every solution is developed with a holistic view of its clinical applicability, technical feasibility, and ethical implications. Examples include federated admission prediction to support real-time bed management and reduce ED boarding, deterioration models to streamline triage and accelerate clinician decision-making, and predictive discharge tools to assist case managers with patient flow coordination. By embedding implementation partners from the outset, FAIR-EC functions as both a data science network and an implementation platform, enabling the alignment of technical innovation with practical workflow improvements across diverse health care systems.

### Implementation and Workflow Impact

A core objective of FAIR-EC, beyond developing predictive models, is to ensure that these models translate into meaningful efficiency gains for clinicians, administrators, and institutional leaders, who are the end users of EC systems. FAIR-EC brings together data scientists, emergency physicians, nursing staff, and hospital leadership in cross-disciplinary teams to co-design projects around pressing operational challenges.

### Communication Channels and Workshops

Scheduled virtual and in-person meetings, when feasible, will ensure ongoing dialogue between all team members. These meetings will serve as forums for progress updates, brainstorming sessions, and resolving any challenges that arise during the collaboration. The FAIR-EC collaboration will organize workshops that bring together participants from various disciplines to share knowledge, discuss emerging trends in EC and data science, and explore the ethical considerations relevant to the project. These workshops will be crucial for maintaining the alignment of the project with the latest scientific and ethical standards.

### Iterative Feedback Mechanisms and Stakeholder Engagement

Iterative feedback mechanisms and the incorporation and assessment of stakeholder input are central to refining our models and tools within the EC context (Figure 2). Prototypes developed will be tested by EC professionals in simulated or real-world settings, and their feedback will be crucial for ensuring that these solutions meet the practical needs of EC settings. Additionally, ethical review panels, including ethicists and social scientists, will periodically review the project’s progress to ensure ethical considerations are thoroughly addressed. These panels will guide necessary adjustments based on the ethical implications of the data science techniques used. This proposed feedback loop will allow for continuous refinement of models based on clinical impacts observed in practice and insights from ethical reviews, ensuring alignment with clinical needs, ethical standards, and societal expectations.

We will also use feedback from various stakeholder groups to inform and enhance the presentation of risk estimates. If different subgroups, such as health care administrative leadership and patients, highlight distinct priorities, we will consider tailoring the presentation to better meet these specific needs. This feedback will be assessed through a second phase of focus groups, which will evaluate the appropriateness and effectiveness of the presentation adjustments. This phase will include discussions and ranking processes to finalize communication options. We plan to conduct 3 focus groups, one for each type of stakeholder, drawing from the same participant pool as the initial phase.

### Governance and Conflict Resolution

The FAIR-EC network operates under a multitiered governance structure designed to balance clinical, technical, and ethical perspectives. A central leadership team coordinates strategic direction and day-to-day activities, supported by an international advisory panel that provides independent guidance. Interdisciplinary working groups are responsible for reviewing project proposals, developing study protocols, and monitoring ongoing activities.

To address conflicts that may arise between data science objectives and clinical perspectives, FAIR-EC uses structured consensus-building methods such as moderated discussions and Delphi processes. If consensus cannot be reached at the working group level, issues are escalated to the leadership team for resolution in consultation with the advisory panel.

Given the network’s global scope, we anticipate differences in regulations, cultural norms, and data governance requirements across sites. Local site leads are responsible for ensuring compliance with regional laws and ethical standards, while FAIR-EC provides overarching principles to align practices. Communication materials and protocols are designed to support multilingual participation, and data harmonization workflows account for both technical heterogeneity and cultural considerations in the interpretation of clinical data. This governance structure ensures that FAIR-EC remains inclusive, ethically grounded, and adaptable to diverse health care contexts.

### Ethical Oversight Structure

Ethical oversight within FAIR-EC is embedded at multiple levels to balance collective accountability with local autonomy.

At a network level, steering and ethics working groups establish overarching ethical principles, provide strategic guidance, promote consistency across projects, and mediate conflicts when they arise, while preserving the decentralized ethos of federated learning.

On the project level, for each project undertaken within FAIR-EC, the project’s authorship team serves as the coordinating body. These teams are responsible for applying FAIR-EC principles in study design and implementation, integrating interdisciplinary expertise, and reviewing project protocols in collaboration with participating sites.

Finally, at the local site level, each participating institution maintains responsibility for compliance with its own IRBs, regulatory requirements, and cultural norms. Local investigators ensure that implementation respects regional laws and governance structures while contributing to the shared ethical standards of the network.

This layered structure ensures that ethical oversight is exercised consistently across the network while remaining sensitive to local contexts and governance requirements, as shown in Figure 2.

### Planned Recruiting Strategy

To strategically expand our network of participating sites, FAIR-EC plans to engage with global and regional EC organizations and conferences. We will present our research goals and preliminary findings at international health care symposiums to attract interest from potential site partners. Additionally, targeted outreach will be conducted to leading hospitals and research institutions known for their innovative approaches to EC. By offering these institutions the opportunity to contribute to and benefit from our federated research model, we aim to ensure a diverse and representative array of sites that can provide valuable data and insights, enhancing the overall impact and applicability of our findings in the global context of EC. Although the protocol primarily targets ED patients, the inclusion of patients is scalable, and we will not limit our scope solely to ED patients, allowing for the expansion of patient categories as this study progresses.

For additional institutions interested in joining the FAIR-EC network, we have established a clear and accessible pathway for collaboration. Interested parties can reach out through a dedicated section on the FAIR-EC project website, which details the collaboration process, including eligibility criteria, expected contributions, and benefits of participation. Institutions can also contact the FAIR-EC administrative team directly via email to express interest and receive guidance on the application process. Furthermore, we will offer informational webinars and question-and-answer sessions to provide prospective sites with a comprehensive understanding of the project’s scope, goals, and collaborative framework. This open and structured approach ensures that any institution, regardless of its location or size, could become part of this transformative research endeavor.

### Multimodal Data

We use data from 2 distinct modalities: structured codified data and unstructured data. Structured data encompasses quantifiable and easily searchable elements such as demographics, diagnosis codes, medications, laboratory tests and results, procedures, and genomic or genetic data (if available). Some social determinants of health are also included in this category, enabling the systematic analysis of trends in diagnoses and treatments (eg, Index of Relative Socio-Economic Disadvantage in Australia [52] and indexes of socioeconomic disadvantage or socioeconomic advantage indices in Singapore [53]). Such variables should be carefully selected and organized to keep track of the socioeconomic status over time.

In contrast, unstructured data provides richer context and nuance. This includes clinical narratives (progress notes, discharge summaries, and radiology reports) that offer insights into patient symptoms and treatments not captured by structured fields. It also covers narrative accounts of social determinants of health, imaging data such as plain radiography and advanced diagnostic imaging (eg, computed tomography and magnetic resonance imaging) for rapid diagnosis, and waveform data (eg, electrocardiograms and electroencephalograms) that reveal real-time physiological responses. These unstructured modalities are indispensable for immediate, accurate medical decision-making in EC, supplementing the foundational insights obtained from structured data.

It is important to note that there may be variations in the availability, format, and definitions of each data modality across different participating sites in the FAIR-EC collaboration. Not all sites have access to the same range of modalities, such as imaging data or detailed genotype data, and even when similar data types are available, they may differ significantly in format and detail depending on regional practices and technological infrastructure. This variation necessitates comprehensive data harmonization efforts proposed in the next section, which are critical to ensure the integrity and comparability of the data across all sites. By allowing for the unique contributions of each site’s available data and addressing discrepancies in data formats systematically, this study maximizes the breadth and depth of its insights while maintaining rigorous standards of data quality and consistency.

### Planned Privacy-Preserving Federated Learning System

A key strategy in our approach is the use of federated learning, which will be adapted from our previous work [54,55]. The federated learning framework allows for the collaborative analysis of data from various sites without the need to centralize data, thus preserving patient privacy and data confidentiality. By distributing the computational tasks across multiple participating institutions, federated learning enables the FAIR-EC study to benefit from the rich diversity of global data while adhering to strict privacy standards. This approach is particularly effective in EC, where the variability of EC practices and outcomes across different regions can be vast.

To ensure broad participation across institutions with varying technical capacities, FAIR-EC adopts a tiered participation model. At one level, high-resource sites are able to run local model training directly on institutional servers, contributing model updates to the central coordinating layer while keeping raw data fully local. For sites with access to institutionally governed cloud environments, participation can be facilitated through the deployment of widely available federated learning toolkits (eg, Flower, TensorFlow Federated, and PySyft), which reduce local infrastructure demands while maintaining institutional oversight of data. In settings without access to cloud resources, containerized deployments (eg, Docker or Kubernetes) provide a lightweight, reproducible environment that can operate on modest local hardware, thereby lowering barriers to entry. This approach preserves flexibility while ensuring comparability across sites, making it possible for institutions with different resource levels to contribute meaningfully to FAIR-EC. By combining centralized coordination of model aggregation with multiple pathways for site-level engagement, the framework enables scalable participation without compromising privacy or creating undue technical burden.

While federated learning avoids direct data sharing, we acknowledge that residual privacy risks remain, including potential gradient or model inversion attacks. To mitigate these risks, FAIR-EC incorporates secure aggregation, differential privacy mechanisms, and gradient clipping. For small sites or rare cohorts, where limited sample size may heighten reidentification risk, we adopt additional obfuscation strategies such as update padding, feature bucketing, and stronger noise injection, with minimum cohort thresholds required before updates are shared. Where thresholds are not met, sites may participate through pooled aggregation or contribute via federated analytics rather than direct model updates. To further reduce disparities in participation, our framework emphasizes communication-efficient algorithms, adaptive update protocols, and lightweight containerized deployments that minimize setup requirements for sites with limited infrastructure. These safeguards collectively ensure that privacy is preserved while enabling fair engagement across institutions with diverse capacities.

### Planned Data Harmonization and Knowledge Network

Participating sites will use common data standards such as the Observational Medical Outcomes Partnership [56], Patient-Centered Outcomes Research Network [57], and Informatics for Integrating Biology and the Bedside [58]. We do not select a single model to ensure flexibility across diverse data environments and infrastructure levels among international sites. These models facilitate the standardization and interoperability of data across different health care systems, enabling a cohesive analysis despite the heterogeneity of the datasets. To address the challenges of data diversity and ensure a unified dataset for analysis, this current study proposes using the established BERT architecture for its proven effectiveness in understanding contextual relationships within text [59-61], and augmenting it with newer transformer models such as GPT-4, for enhanced data harmonization and metadata creation [62]. This dual approach allows us to leverage the robust foundational capabilities of BERT while benefiting from the advanced contextual processing powers of recent transformers. To integrate data from different common data models, these tools will be instrumental in translating disparate data elements into a standardized format, ensuring consistency and comparability across the datasets. Notably, in some countries, LLM workflows have been actively implemented to extract data from electronic medical records for research purposes, though these technically feasible implementations still await regulatory approval.

To effectively manage the diverse multimodal and multisource datasets, we propose to implement sophisticated algorithms and tools designed to aggregate and process data from various modalities. Specifically, we are using our previously developed semantic learning approaches [63,64], state-of-the-art multimodal deep learning architectures [65], and contrastive learning [66] to derive meaningful representations from these large datasets without the need for phenotypic (labeled) data, which is often scarce in health care settings. This approach enables us to harness the full potential of available data, improving our ability to identify and analyze complex patterns associated with outcomes that are inherent in the multifaceted nature of health data. We focus on semantic integration, creating cohesive semantic spaces that allow for seamless interaction across different data modalities, such as structured data, unstructured clinical narratives, and genotype data.

As part of our broader strategy for data harmonization and representation learning, we will develop a GCN that incorporates a prototype graph [67]. A prototype graph will be developed to serve as a conceptual model to define and visualize the fundamental relationships and interactions among various dataset elements, such as multimodal covariates, risk factors, and health outcomes, which is crucial for mapping complex interdependencies. By using the prototype graph, we can precisely characterize and understand the intricate relationships within our data, informing the development and refinement of our association studies’ predictive models tailored to EC settings. We plan to train the GCN using a combination of publicly available datasets from participating sites that have been annotated by a team of domain experts and data scientists. These datasets will encompass a diverse range of EC scenarios to ensure robust feature representation. Labeling will be overseen by clinical professionals to maintain accuracy and relevance. To validate the GCN’s generalizability, we will conduct cross-validation studies across multiple participating sites, comparing performance metrics against baseline models to ensure it effectively models the relationships among data from various settings.

In addition, we recognize the role of vendor-facilitated platforms such as Epic Cosmos (Epic Systems Corporation), which demonstrate the potential for large-scale data aggregation within Epic-using institutions. However, reliance on such platforms alone would be restrictive, as not all participating sites use Epic and many already operate research infrastructures based on other data models. We therefore position Epic Cosmos as one option within a broader ecosystem, while ensuring that FAIR-EC remains flexible and inclusive across diverse EHR systems and international contexts.

For semantic alignment across sites, we supplement these mappings with ontology bridging (eg, Unified Medical Language System and SNOMED CT) and transformer-based methods for harmonizing unstructured text. This layered approach allows local flexibility while preserving a shared analytical layer, enabling federated analyses that are both robust and adaptable to the diverse data environments represented in FAIR-EC.

### Planned Data Analytics and Model Development

**Figure 3.**
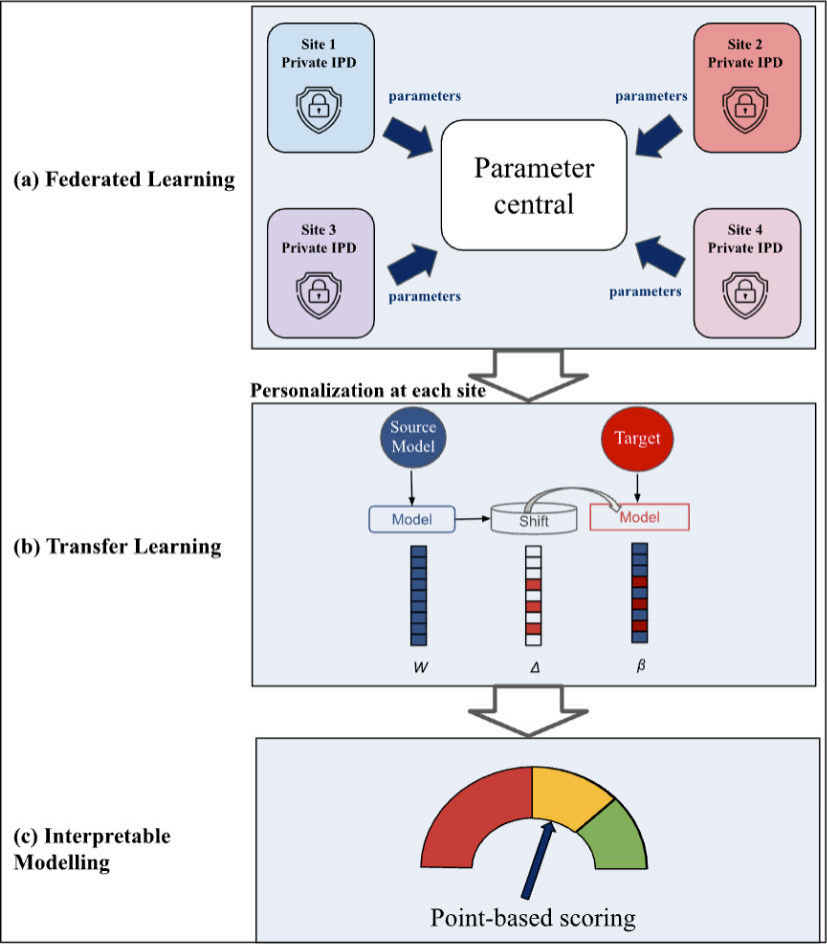
Framework for model development under federated learning: transfer learning and interpretable modeling. IPD: individual patient data.

Our approach to data analysis and model development in FAIR-EC is to be adaptive, allowing the application of different and emerging techniques based on the specific needs of each project. Under the privacy-preserving federated learning system, our general approach is structured around two main modules: transfer learning for local adaptation, and point-based scoring systems to enhance interpretability, with a multimodal transformer as the backbone, adept at managing diverse data across various modalities, sample rates, and formats, even when unaligned (as illustrated in Figure 3).

### Federated Transfer Learning and Negative Transfer Mitigation

Transfer learning involves taking a preexisting model developed for one task or from one population and adapting it for another related task or population [68]. In the context of EC, this includes leveraging knowledge and patterns learned from one health care system or dataset to improve or tailor models for another, with particular attention to addressing disparities in underrepresented groups. Transfer learning can function in two distinct ways: (1) as a model calibration step following federated learning, where it fine-tunes global models to local contexts, and (2) as an independent approach, particularly useful in low-resource settings. In such cases, researchers can directly leverage knowledge from published studies to adapt and enhance models for their new studies, even when they cannot establish a suitable collaboration in a short time. A notable example from our previous work illustrates how we address demographic disparity in the presence of class imbalance, by leveraging recent advances in imbalance learning, transfer learning, and federated learning [69].

Transfer learning can extend model utility to sites with limited data, but its effectiveness depends strongly on domain similarity. Applying models across heterogeneous health care systems raises risks of negative transfer and bias amplification. To address these concerns, FAIR-EC adopts a federated transfer learning strategy that draws from multiple potential source sites rather than relying on a single external dataset. By evaluating similarity across candidate source domains, the framework preferentially borrows knowledge from institutions with population and practice patterns most aligned with the target site. When similarity is low, adaptation techniques are used to recalibrate models, and contributions from dissimilar sites are down-weighted or excluded to avoid negative transfer.

### Equity Considerations, Ethical Implications, and Engagement Plan

Ensuring that EC models are equitable also necessitates a rigorous ethical framework guiding the collection, use, and interpretation of EHR data. This framework should prioritize transparency, accountability, and inclusiveness in model development and deployment, particularly regarding how data are used and how decisions based on model predictions impact patient care across different populations. The commitment to these ethical principles not only enhances the credibility and acceptability of association studies’ predictive models but also ensures that these innovations contribute positively to public health goals, reinforcing trust among all stakeholders, including patients, health care providers, and health care administrative leadership. Active engagement of stakeholders is crucial for developing models that are technically sound, ethically grounded, and practically relevant. To this end, we will hold regular workshops and feedback sessions with clinicians, patients, and policymakers to integrate diverse perspectives directly into the development and implementation processes.

### Planned Evaluation Strategy

We propose a comprehensive evaluation strategy designed to rigorously assess the performance and impact of the developed models, which not only assesses the technical performance of the developed models but also critically examines their ethical implications, fairness, and real-world utility. By rigorously benchmarking against traditional tools, comparing with black box algorithms, and focusing on fairness and equity, the FAIR-EC collaboration seeks to pave the way for the responsible and effective use of data science in EC.

### Benchmarking Against Current State-of-the-Art Standards in EC

A crucial aspect of the evaluation strategy involves benchmarking the performance of the developed data science models against traditional EC tools currently in use. Specifically, we will compare our models to traditional clinical practice guidelines, which are driven by evidence and expert opinion, as well as to current decision support tools integrated within EHRs. This comparison will focus on various metrics such as accuracy, efficiency, and AUROC. The objective is to demonstrate tangible improvements and advantages of adopting advanced data science techniques in EC settings, thereby justifying the transition from traditional practices to more innovative, data-driven approaches.

### Comparison With Black Box AI Algorithms

This study will undertake a critical comparison between the interpretable models developed within the FAIR-EC framework and existing “black box” algorithms, which are known for their advanced and sophisticated architectures but lack transparency. While black box models often demonstrate superior predictive power, we aim to show that incorporating value-added principles such as transparency, fairness, and interpretability can achieve noninferior performance [70]. This comparison will highlight the ethical and practical advantages of interpretable models, emphasizing their role in clinical decision-making tools that clinicians can trust and understand. By demonstrating comparable performance alongside enhanced explainability, we aim to facilitate the broader adoption of these models in EC practices, ensuring they align with both ethical considerations and clinical needs.

### Fairness and Equity Assessment

This study will use advanced methodologies specifically designed to assess algorithmic fairness across sociodemographic groups, ensuring that the models do not perpetuate existing biases or create new disparities in EC. This rigorous assessment will involve a detailed analysis of the models’ performance and outcomes across different patient populations, with a focus on identifying any discrepancies or biases in predictions or recommendations. Methods such as disparity impact analysis, fairness metrics (eg, Canadian Index of Multiple Deprivation [71]) and equity rubric lists (eg, PROGRESS Plus) [72] will be used to systematically evaluate how algorithms perform across various groups defined by age, gender, ethnicity, socioeconomic status, and other relevant factors, depending on the data available at each site.

To further enhance the evaluation of fairness and equity, we will engage a diverse group of stakeholders—including patients, health care providers, health care administrators, population health scientists, and economists—both before and after model deployment. This comprehensive approach ensures that our models are developed and continuously improved with input from those most impacted by their implementation. We will also identify and use specific indices, such as access to care and health outcome disparities, to adjust and calibrate the model for greater equity throughout its lifecycle.

### Potential Bias Mitigation Strategies

Recognizing the critical importance of addressing and mitigating bias, this study will explore and implement a range of strategies designed to ensure the fairness and equity of the developed models. These strategies may include rebalancing datasets, using algorithmic fairness techniques, and incorporating feedback loops that allow for the continuous monitoring and adjustment of models based on real-world performance data. By actively engaging in bias mitigation, the FAIR-EC study aims to set a new standard for the development and deployment of equitable data science solutions in EC.

### Ethical Considerations

As this paper describes a research protocol for a federated, multi-institutional collaboration, no individual patient-level data are shared centrally, and no centralized analyses of identifiable data have been conducted to date. As of the time of writing, ethics review board (IRB) applications are under preparation or review at participating institutions for their respective data contributions and analyses. Each site in the FAIR-EC network is responsible for obtaining approval from its own ethics board before engaging in data analysis or federated learning. The Duke University site operates under the oversight of the Duke Health IRB (Pro00120917), which covers federated infrastructure development and use of deidentified EHR data for modeling. On the other hand, at the coordinating site at Duke-NUS Medical School (Singapore), this study operates under the ethics policies of the NUS-IRB-2021-766. Under the National Healthcare Group Domain Specific Review Board, it adheres to SingHealth’s Centralised Institutional Review Board Ref: 2021/2122.

This protocol paper does not report any direct analysis of patient-level data and is thus exempt from centralized ethics board review under institutional policy (Duke IRB SOP 1.2; National Healthcare Group Domain Specific Review Board guidelines on multisite research, version 12). Future analyses will be conducted only after appropriate IRB or ethics board approval is obtained at each participating site, in compliance with regional and institutional guidelines for human participants research.

Beyond technical safeguards and fairness metrics, the ethical foundation of FAIR-EC centers on the responsible stewardship of innovation in high-stakes, time-sensitive clinical environments. EC often involves rapid decisions under uncertainty; integrating AI must not exacerbate power imbalances, erode clinical autonomy, or marginalize vulnerable populations. FAIR-EC explicitly rejects approaches that prioritize performance over transparency or that rely on opaque, nongeneralizable models. Instead, we emphasize deliberative governance, participatory design, and contextual adaptability across geographies. Our ethical stance informs not only model development but also decisions about deployment, data sharing, and global collaboration, ensuring that FAIR-EC remains accountable not just to regulators or researchers but to the patients and communities it aims to serve.

As this protocol involves secondary analysis of routinely collected, deidentified EHR data, individual informed consent is waived at participating sites in accordance with local IRB determinations. Importantly, no identifiable patient data will leave institutional boundaries; only aggregated model parameters are shared during federated learning, and all data are deidentified in accordance with applicable regulations (eg, Health Insurance Portability and Accountability Act, and Personal Data Protection Act) [73,74]. As ED populations inherently include vulnerable populations, the FAIR-EC protocol addresses these considerations through fairness-aware modeling that evaluates performance across demographic subgroups to prevent algorithmic bias. As this is a protocol paper, demographic characteristics will be reported in future publications describing model development and validation. No compensation is provided to patients, as this study involves retrospective analysis of existing records with no direct patient contact. All future analyses will adhere to the WMA (World Medical Association) Declaration of Helsinki.

### Current Status and Planned Projects

The FAIR-EC study has made significant strides, starting with establishing a comprehensive network of participating institutions across the globe. These efforts are not presented as formal analyses under this protocol, but rather as examples of ongoing and planned work that align with the FAIR-EC framework. Specific modeling strategies, evaluation metrics, and validation procedures will be introduced in project-level protocols or subsequent publications.

### Participating Institutions and Teams

#### Overview

**Figure 4.**
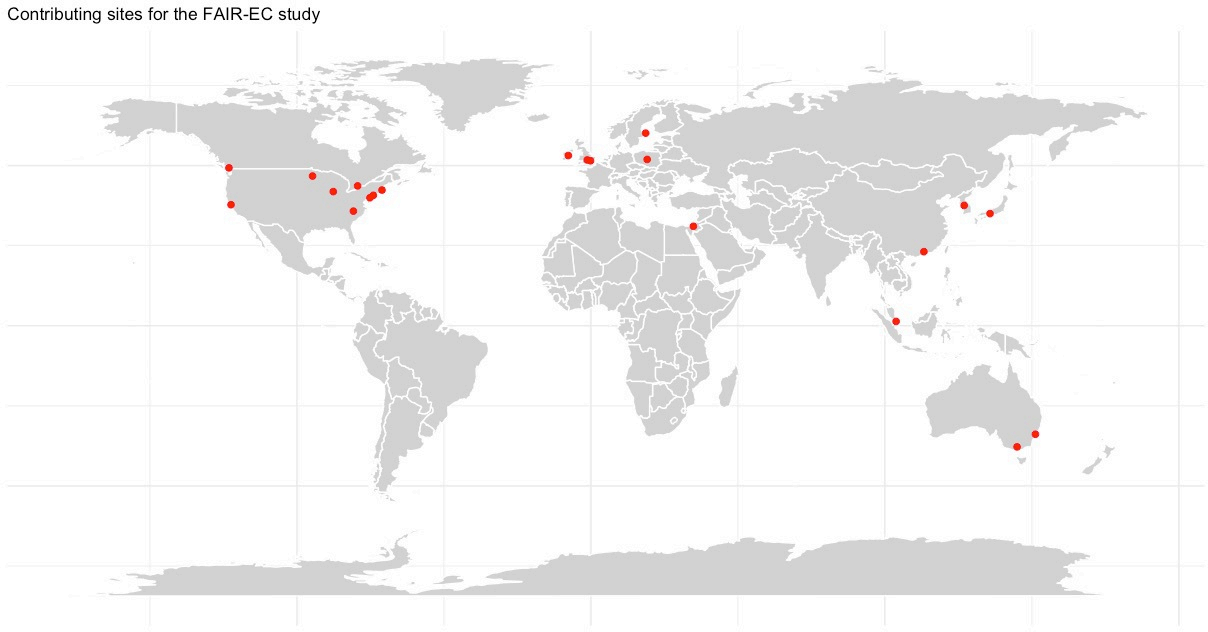
Global collaboration in the FAIR-EC Initiative, more than 20 contributions from multinational research networks across North America, Asia, Europe, and the Middle East. FAIR-EC: Fair, Accountable, Interpretable, and Responsible-Emergency Care.

The FAIR-EC collaborative framework has already facilitated a range of projects targeting association and predictive modeling, efficiency optimization, and fairness in EC delivery. The initiative’s global reach, from North America and Asia to Europe, and now the Middle East, sets a strong foundation for impactful research aimed at improving emergency medicine practices and patient outcomes worldwide (Figure 4).

#### North America

The initiative is bolstered by contributions from institutions in the United States, including Duke University, Columbia University, Harvard Medical School’s Brigham and Women’s Hospital, Cornell University, Northwestern University, University of Minnesota, and University of California San Francisco. These institutions have made available substantial patient records, with Duke University alone contributing data on more than 400,000 patients. In addition, the University of British Columbia and the University of Toronto represent our Canadian cohorts.

#### Asia

This study’s Asian contingent features Singapore’s Duke-National University of Singapore Medical School, Kandang Kerbau Women’s and Children’s Hospital, National University Health System, Lee Kong Chian School of Medicine, and Agency for Science, Technology and Research, with Singapore General Hospital providing access to over 1.7 million patient records. South Korea is represented by Samsung Medical Center, Yonsei University, and Hallym University Medical Center, which add additional patient records to this study’s database. Japanese patients are also represented by Kyoto University. The First Affiliated Hospital of Sun Yat-Sen University extends this study’s reach into China, enriching the diversity of data sources.

#### Europe, Australia, and the Middle East

In Europe, the University of Oxford and King’s College London in the United Kingdom, University College Dublin in Ireland, Karolinska Institutet and Karolinska University Hospital in Sweden, and Provincial Specialist Hospital in Poland contribute their expertise, while Monash University and the University of Sydney extend this study’s reach to Australia. Notably, the University of Haifa in Israel represents the Middle East, adding to this study’s geographical and cultural diversity. This inclusion ensures that the FAIR-EC study encompasses a wide range of health care systems and patient demographics, offering a comprehensive perspective on EC practices worldwide.

## Results

**Figure 5.**
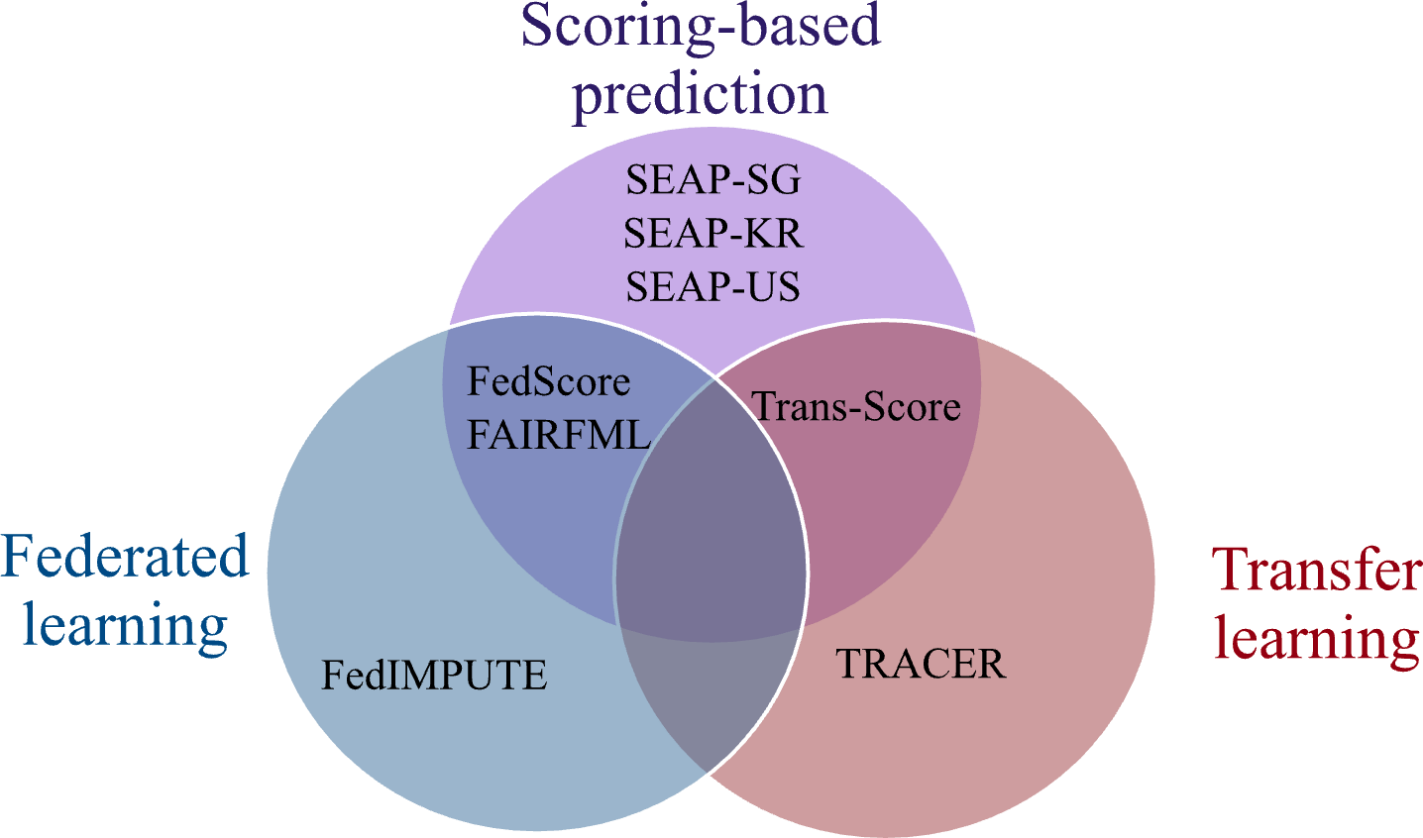
Ongoing projects enabled by the FAIR-EC study. FAIR-EC: Fair, Accountable, Interpretable, and Responsible-Emergency Care; SEAP-KR: Score for Emergency Admission Prediction-Korea; SEAP-SG: Score for Emergency Admission Prediction-Singapore; SEAP-US: Score for Emergency Admission Prediction-United States.

All individual sites participating in this initiative (if their data are provided for collaboration) will obtain the necessary ethical approvals to ensure that the research complies with local and international ethical standards. Since its inception in September 2022 (funded under the Duke/Duke-NUS Collaboration Pilot Grant period September 1, 2022, to August 31, 2023), FAIR-EC has initiated a series of projects that exemplify the practical application of advanced data science techniques in EC, grounded in the principles of international collaboration (Figure 5). Among these, a federated scoring system is being developed to enable cross-institutional association and predictive modeling without compromising patient data privacy, illustrating the potential of federated learning in health care [33-35]. For this protocol submission (January 2026), participating sites had identified retrospective EC datasets totaling >2 million records, including US cohorts (eg, Duke Health ED encounters from calendar year 2019 across 3 hospitals for admission prediction and federated-imputation pilot work) and large regional datasets in Asia (eg, Singapore General Hospital with >1.7 million patient records), as well as additional international partners. As of January 2026, cross-site data harmonization and federated workflow implementation had been initiated, with local IRB or ethics review at participating sites in progress. Concurrently, a project is underway to evaluate the generalizability of the inpatient admission prediction models originally developed in Singapore across multiple international sites, with initial cross-site benchmarking analyses expected to be submitted in mid-late 2026. This initiative assesses the accuracy of the Score for Emergency Admission Prediction-Singapore and its adaptations, the Score for Emergency Admission Prediction-Korea and the Score for Emergency Admission Prediction-United States, in diverse health care settings. Another notable effort involves the use of transfer learning to adapt these Singapore-developed models for use at Duke University Hospital, tailoring the models to better fit the specific patient demographics and care practices of the hospital environment (FedScore [48,49,75] and FairFML [33]). Additionally, the current study is exploring dynamic transfer learning to ensure that these association and predictive models can be continuously updated over time, maintaining their relevance and accuracy in the face of evolving health care practices and patient populations (TRACER and Trans-Score, WO2025221209A1 [76]). Moreover, a federated-transfer imputation method has been proposed to handle missing data across different datasets, further enhancing the robustness and applicability of the federated models (FedIMPUTE). Collectively, these projects reflect the types of collaborative efforts enabled by the FAIR-EC infrastructure. While not part of a single analytic protocol, they demonstrate the feasibility of federated modeling, transfer learning, and harmonized data strategies across sites. Project-level papers are planned for submission in 2027, aligned with the completion of harmonized extracts, federated analyses, and site-specific ethics milestones.

## Discussion

### Principal Findings

FAIR-EC is dedicated to integrating ethical AI principles into the development and application of data science techniques within EC. By leveraging advanced methodologies such as federated learning and transfer learning, this study aims to enhance association and predictive modeling, improve patient stratification, and ensure equitable care across diverse health care settings. The significance of FAIR-EC lies in its pioneering approach to addressing complex challenges in EC through data science. By focusing on the ethical application of AI [14], this current study not only seeks to improve patient outcomes but also to set a new standard for the responsible use of technology in health care. The international and interdisciplinary collaboration at the heart of FAIR-EC underscores the global relevance of its mission, highlighting the universal need for improved EC solutions.

FAIR-EC boasts a broad geographical representation and a rich diversity of data, enabling a comprehensive analysis of EC practices and outcomes. The adoption of federated learning and other privacy-preserving techniques ensures the ethical handling of patient data, fostering trust and collaboration among participating institutions. Moreover, the collaboration’s focus on transfer learning facilitates the adaptation of models across different settings, enhancing their applicability and impact.

The variability in data quality and completeness across sites poses challenges to model development and validation. Additionally, the complex nature of health care data and the evolving landscape of emergency medicine require ongoing adjustments to modeling approaches. At present, the network is primarily composed of well-resourced sites, limiting our capacity to engage lower-resource settings. To address these issues, FAIR-EC plans to establish localized data analytical teams at each site, enhancing our federated model with site-specific expertise and resources, and exploring mentorship mechanisms to support future partners in less-resourced environments. Future efforts will also focus on developing more robust mechanisms for model evaluation and on exploring new areas where data science can contribute to EC, such as operational efficiency and patient experience.

In assessing the use of multimodal data in EC, it is crucial to acknowledge the inherent biases in data collection. Availability and depth of data—ranging from structured codified data to imaging and genotype data—vary significantly worldwide. Some regions have advanced systems capable of extensive data generation, while others might lack these resources. This disparity not only impacts data quality but also the applicability of advanced data-driven methodologies, potentially reinforcing health inequities.

Implementing challenges include technological integration, workflow understanding and integration, staff training, and the potential resistance from health care professionals accustomed to traditional methods of care delivery. Addressing these issues would require comprehensive education and demonstration of the model’s efficacy and benefits.

The pioneering projects initiated by the FAIR-EC study facilitate further innovation in EC. By leveraging the feasibility and effectiveness of federated learning, transfer learning, and dynamic model updating in real-world health care settings, these projects lay the groundwork for future research endeavors. As these initial projects validate the use of advanced data science techniques across different geographies and health care systems, they are expected to inspire a broader adoption of these approaches, facilitating more personalized, efficient, and equitable EC solutions. Furthermore, the successful implementation and outcomes of these projects will likely attract participation from additional sites and foster stronger interdisciplinary collaborations, expanding the scope and impact of FAIR-EC. This momentum is anticipated to generate a self-reinforcing cycle of innovation, where each success story paves the way for new questions, hypotheses, and projects, ultimately driving continuous improvement in EC practices worldwide.

To ensure that these efforts translate into tangible scientific contributions, the FAIR-EC leadership has established a coordinated publication pipeline, with writing teams, target journals, and timelines prespecified for each project. Lead authors are assigned at project kickoff, and each publication is expected to include investigators from multiple institutions to reflect FAIR-EC’s collaborative spirit. Dedicated paper development workshops, internal peer review processes, and milestone tracking within the collaboration platform will support timely and high-quality dissemination. The first set of papers is expected to be submitted in early to mid-2026, with additional publications planned as subsequent analyses mature.

### Conclusion

FAIR-EC is a pioneering initiative that applies data science ethically, equitably, and effectively in EC. By leveraging federated learning, transfer learning, and interpretable AI, FAIR-EC establishes a scalable and privacy-preserving framework for collaborative model development across diverse health care settings. While challenges related to data variability, health care system complexity, and cross-institutional harmonization persist, this initiative lays a strong foundation for ongoing innovation in global EC and medicine.

Moving forward, FAIR-EC commits to three key promises: (1) expanding the network by integrating more diverse institutions and health care systems to improve model generalizability and equity; (2) enhancing methodological rigor through continuous refinement of federated, transfer, and explainable learning approaches to improve clinical utility; and (3) ensuring real-world impact by translating research findings into actionable clinical tools that empower emergency physicians, policymakers, and health care providers.

With these commitments, FAIR-EC aspires to set a new global standard for fair, accountable, and interpretable AI applications in EC—bridging the gap between cutting-edge data science and real-world patient care.

## Supplementary material

10.2196/74202 Multimedia Appendix 1 Motivations behind the FAIR-EC framework. FAIR-EC: Fair, Accountable, Interpretable, and Responsible-Emergency Care.

## Abbreviations

AI: artificial intelligence
AUROC: area under the receiver operating characteristic curve
BERT: Bidirectional Encoder Representations From Transformers
EC: emergency care
ED: emergency department
EHR: electronic health record
FAIR-EC: Fair, Accountable, Interpretable, and Responsible-Emergency Care
GCN: graph convolutional network
*ICD*: *International Statistical Classification of Diseases*
IRB: institutional review board
LLM: large language model
PROGRESS: Place of Residence; Race, Ethnicity, Culture, and Language; Occupation; Gender or Sex; Religion; Education; Socioeconomic Status; Social Capital
SNOMED CT: Systematized Nomenclature of Medicine – Clinical Terms
WMA: World Medical Association
