# Supplementary material for: Building a Global Research Network for Fair, Accountable, Interpretable, and Responsible AI in Emergency Care: Protocol for a FAIR-EC Study
Source: JMIR Res Protoc. 2026 Aug 4;15:e74202. doi: 10.2196/74202 (PMC13439576; doi:10.2196/74202)
Supplement: Supplementary file 1 [file resprot-v15-e74202-s001.docx]

As mentioned in the Introduction of our main text, our proposed Fair, Accountable Interpretable and Responsible (FAIR) Framework was developed to fully integrate ethical AI principles in the Emergency Care (EC) setting. To accomplish this, we have reviewed the current AI and EC scene, understood the problems faced by clinicians and summarised barriers to their implementation. In this section, we elaborate on the importance of our choice of each ethical principle, and the challenges each principle faces in the field of EC.

## Fair

Fairness in EC is the principle that ensures all members of society can receive an appropriate quality of care befitting the severity of their condition, irrespective of their background, race, gender, socioeconomic status, or age^1,2^. AI-assisted decision-making can provide unbiased, equitable life-saving access to treatment in the fastest manner possible^2^. Fair treatment builds trust between healthcare providers and the public, as people rely on these providers during their most vulnerable moments^3^.

However, achieving fairness can be challenging due to the dynamic and unpredictable nature of emergencies, which often require constant reallocation and the reshuffling of resources to meet the competing needs of various patients^4^. Implicit human-based biases could also lead to certain biases occurring consciously or unconsciously^5^. Furthermore, time constraints during triaging could pose the risk of hasty judgment and oversight^4^.

## Accountable

Accountability ensures that healthcare providers maintain the highest standard of care for all their patients, leading to safer patient outcomes. Having accountability as a cornerstone emphasizes that all healthcare providers are responsible for their decisions and are held accountable for the outcome of their care^6^. By integrating AI tools, we can enhance accountability through more transparent, data-driven recommendations whilst documenting the rationale behind clinical decisions^2^. Consequently, this drives improvements in the quality of care experienced by patients, fostering trust so that patients and families know that they are in good hands^7^.

Notwithstanding, maintaining accountability in EC is difficult due to the life-and-death nature of services provided in the emergency department (ED). The fast-paced environment with the involvement of large, multidisciplinary teams increases the likelihood of errors, leading to suboptimal patient outcomes^8^.

## Interpretable

Interpretability in EC is crucial for refining the decision-making process in healthcare. On one end, it gives patients the right to receive and understand information about their diagnosis, treatment options and costs. This transparency ensures patients are better positioned to make more informed decisions. On the other side of the spectrum, enhanced interpretability means physicians can immediately interpret and target the most pressing issue patients face in complex medical situations, allowing them to circumvent the information overload when a patient’s vitals are read. By making information more interpretable to clinicians, they can methodologically target the most pressing needs of a patient, which might otherwise be obscured by the sheer volume of information synthesized during an emergency^9^. AI-driven solutions play a pivotal role in improving transparency and interpretability by processing large amounts of data, identifying relevant patterns and presenting information to patients and clinicians in the most digestible manner^2^.

However, ensuring interpretability in EC can be challenging due to the inherent complexity of medical information, coupled with the time-sensitive nature of emergencies^8^.

## Responsible

Responsibility is one of the core ethical practices within the medical field. Healthcare workers must work in the best interests of their patient’s well-being, ensuring the highest quality of care possible^10^. This responsibility transcends medical treatment, but also encompasses effective communication, the decision-making process and prioritizing patient safety^10^. By prioritizing responsibility, fosters a culture of accountability and continuous improvement in the ED. With the integration of AI, we can further enhance responsibility through real-time data-driven insights, which enable clinicians to make better-informed decisions, thereby optimizing patient care^2^.

However, being responsible in the fast-paced, high-pressure environment of EC can be challenging. Physicians can be put in the position of treating multiple patients, with limited resources and a time crunch^8^. All these factors result in stress and fatigue, leading to possible sub-optimal decision-making and compromised responsibility.

Overall, by integrating the various ethical principles of fairness, accountability, interpretability and responsibility into EC, this FAIR framework seeks to build trust in the healthcare system, reduce health disparities and enhance overall public health.

## References in Supplementary Materials

1.	Queralt-Rosinach, N., Kaliyaperumal, R., Bernabé, C.H., Long, Q., Joosten, S.A., van der Wijk, H.J., Flikkenschild, E.L.A., Burger, K., Jacobsen, A., Mons, B., et al. (2022). Applying the FAIR principles to data in a hospital: challenges and opportunities in a pandemic. J. Biomed. Semantics *13*, 12.

2.	Inau, E.T., Sack, J., Waltemath, D., and Zeleke, A.A. (2023). Initiatives, Concepts, and Implementation Practices of the Findable, Accessible, Interoperable, and Reusable Data Principles in Health Data Stewardship: Scoping Review. J. Med. Internet Res. *25*, e45013.

3.	Lauridsen, S. (2020). Emergency care, triage, and fairness. Bioethics *34*, 450–458.

4.	Gorick, H. (2022). Factors that affect nurses’ triage decisions in the emergency department: a literature review. Emerg. Nurse *30*, 14–19.

5.	Dehon, E., Weiss, N., Jones, J., Faulconer, W., Hinton, E., and Sterling, S. (2017). A Systematic Review of the Impact of Physician Implicit Racial Bias on Clinical Decision Making. Acad. Emerg. Med. *24*, 895–904.

6.	Rubio-Navarro, A., Garcia-Capilla, D.J., Torralba-Madrid, M.J., and Rutty, J. (2019). Ethical, legal and professional accountability in emergency nursing practice: An ethnographic observational study. Int. Emerg. Nurs. *46*, 100777.

7.	Zhang, Z., Kashyap, R., Su, L., Liu, N., and Meng, Q. (2024). Clinical Application of Artificial Intelligence in Emergency and Critical Care Medicine, Volume IV (Frontiers Media SA).

8.	Schilling, S., Armaou, M., Morrison, Z., Carding, P., Bricknell, M., and Connelly, V. (2022). Understanding teamwork in rapidly deployed interprofessional teams in intensive and acute care: A systematic review of reviews. PLoS One *17*, e0272942.

9.	Blackburn, J., Ousey, K., and Goodwin, E. (2019). Information and communication in the emergency department. Int. Emerg. Nurs. *42*, 30–35.

10.	Lavoie, B., Bourque, C.J., Côté, A.-J., Rajagopal, M., Clerc, P., Bourdeau, V., Ali, S., Doyon-Trottier, E., Castonguay, V., Fontaine-Pagé, É., et al. (2022). The responsibility to care: lessons learned from emergency department workers’ perspectives during the first wave of the COVID-19 pandemic in Canada. CJEM *24*, 482–492.
